# Supplementary material for: In situ analysis of nanoparticle soft corona and dynamic evolution
Source: Nat Commun. 2022 Sep 14;13:5389. doi: 10.1038/s41467-022-33044-y (PMC9474507; doi:10.1038/s41467-022-33044-y)
Supplement: Supplementary file 1 — Supplemental Information [file 41467_2022_33044_MOESM1_ESM.pdf]

# Supplementary Information

# In situ Analysis of Nanoparticle Soft Corona and Dynamic Evolution

Didar Baimanov[1,2,3,4,5,#], Jing Wang[3,#], Jun Zhang[2,4], Ke Liu[1,5], Yalin Cong[1,4,5], Xiaomeng Shi[3], Xiaohui Zhang[3], Yufeng Li[1,5], Xiumin Li[1,5], Rongrong Qiao[1,5] Yuliang Zhao[1,4,6,7], Yunlong Zhou[2,4,8,*], Liming Wang[1,3,4,5,*], and Chunying Chen[1,4,6,7,*]

[1] CAS Key Laboratory for Biomedical Effects of Nanomaterials and Nanosafety, CAS Center for Excellence in Nanoscience, Institute of High Energy Physics & National Center for Nanoscience and Technology of China, Chinese Academy of Sciences, Beijing 100049, P. R. China

[2] Zhejiang Engineering Research Center for Tissue Repair Materials, Wenzhou Institute, University of Chinese Academy of Sciences, Wenzhou 325000, Zhejiang, P. R. China

[3] State Key Laboratory of Natural and Biomimetic Drugs, School of Pharmaceutical Sciences, Peking University, Beijing 100191, P. R. China

[4] University of Chinese Academy of Sciences, Beijing 100049, P. R. China

[5] CAS-HKU Joint Laboratory of Metallomics on Health and Environment & National Consortium for Excellence in Metallomics, Institute of High Energy Physics, Chinese Academy of Sciences, Beijing 100049, P.R. China

[6] The GBA Research Innovation Institute for Nanotechnology, Guangzhou 510700, Guangdong, P. R. China

[7] Research Unit of Nanoscience and Technology, Chinese Academy of Medical Sciences, Beijing 100730, P. R. China

[8] Oujiang Laboratory, Zhejiang Laboratory for Regenerative Medicine, Vision and Brain Health, Wenzhou 325001, P. R. China

[#] These authors contributed equally: Didar Baimanov, Jing Wang

[*] Correspondence and requests for materials should be addressed to: Y.Z. (zhouyl@ucas.ac.cn), L.W. (wangliming@ihep.ac.cn) or C.C. (chenchy@nanoctr.cn)

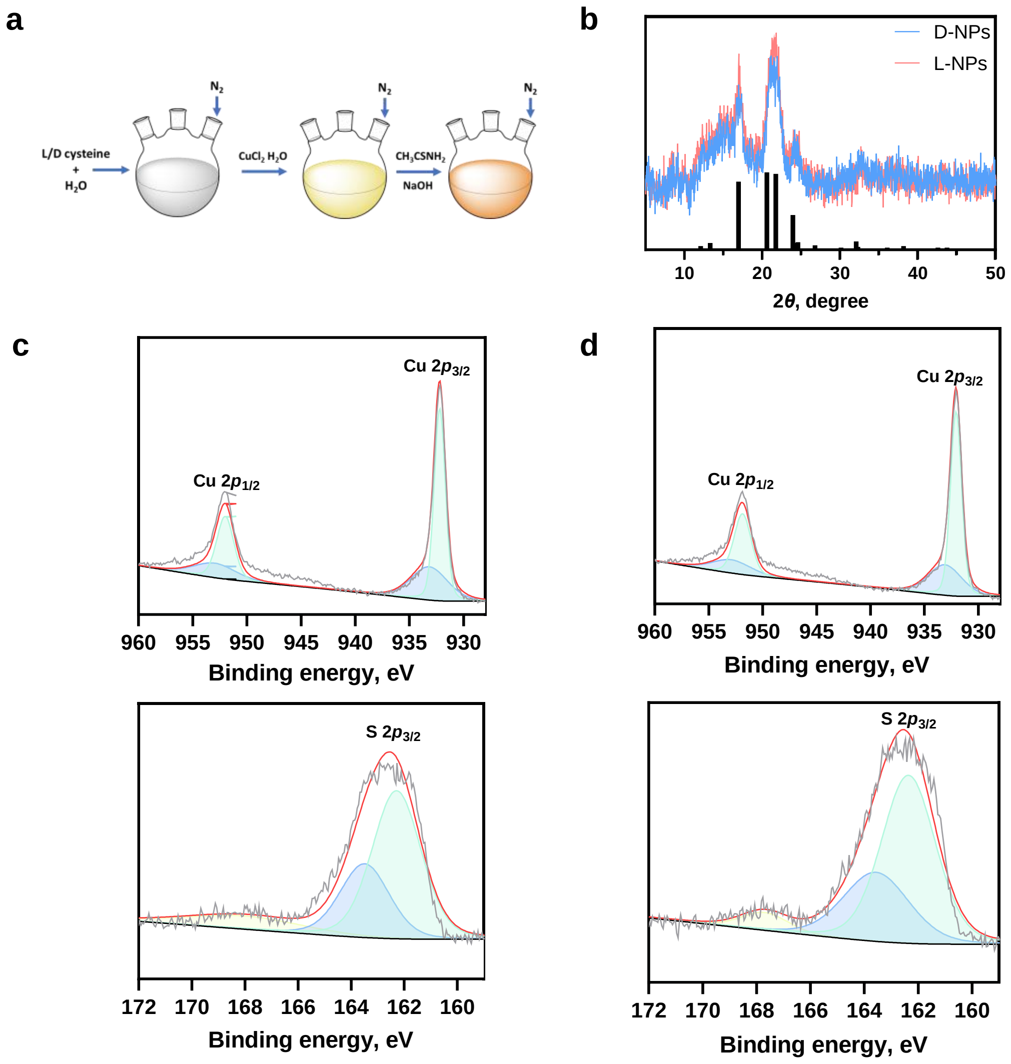


**Supplementary Figure 1. Characterization of D- / L- NPs.** a) Schematic illustration of the chiral NPs synthesis. b) XRD pattern of chiral NPs. The crystal structure of chiral NP is hexagonal $Cu_2S$ [JCPDS cards, No. 26-1116]. Their characteristic peaks (102), (110), (103), and (112) are positioned at 17, 21, 22, and 24 degrees, respectively. c, d) XPS spectra of Cu (2*p*) and S (2*p*) of D-NPs (c) and L-NPs (d), respectively. The binding energy of Cu $2p_{3/2}$ and Cu $2p_{1/2}$ were located at 932.1 eV/ 951.5 eV and 933.0 eV/ 952.9 eV representing the Cu (I, light green) and Cu (II, light blue), respectively. The binding energy of S $2p_{3/2}$ peaks at 162.28 eV (light green) and 163.48 eV (light blue) were identified as two S components from cysteine ligand and $Cu_2S$ NP. A slight peak at 167.7 eV (light yellow) might be the oxidation of nanoparticles during the drying process Source data are provided as a Source Data file.

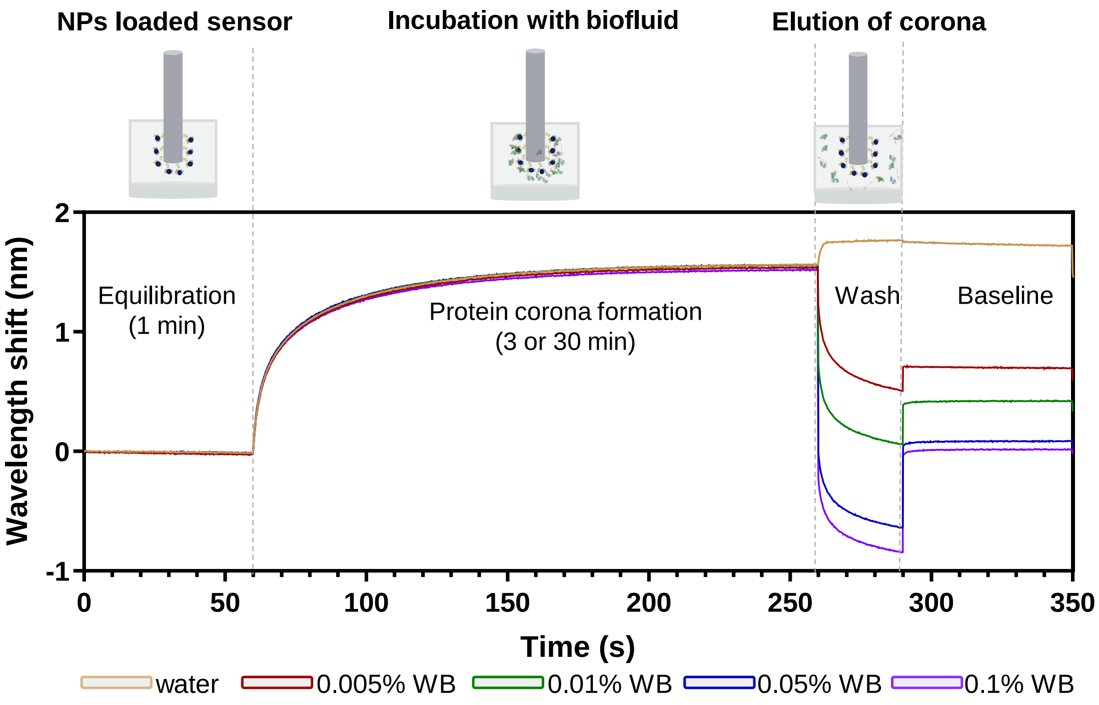


**Supplementary Figure 2. Schematic workflow of the isolation step by BLI-based Fishing method.** Chiral NPs loaded biosensors are firstly equilibrated into buffer solution for 1 min with a further introduction to the biological fluid for 3 min or 30 min. Real-time monitoring for the formation (3 min) of the protein coronas on the surface of chiral NPs is shown. Washing buffer (WB) at different concentrations is used for the isolation of the corona proteins from the chiral NPs-loaded biosensors. 0.005% of WB and 0.1% of WB are used to isolate soft corona and hard corona, respectively. The shift (Δλ) difference of the final baseline and equilibrium steps indicates that chiral NPs remain on the sensor after introducing into the WB. During the adsorption process, the adsorption of proteins increases with time to reach an equilibrium. During the washing step, the proteins can be partly or fully eluted by WB at different concentrations. The NPs for the control (washed by pure water) remain on the sensor, while the signals for NP-protein complex in WB do not decrease at the final baseline compared to the signals at the equilibrium curve, suggesting a stable condition of NPs on the sensor. Source data are provided as a Source Data file.

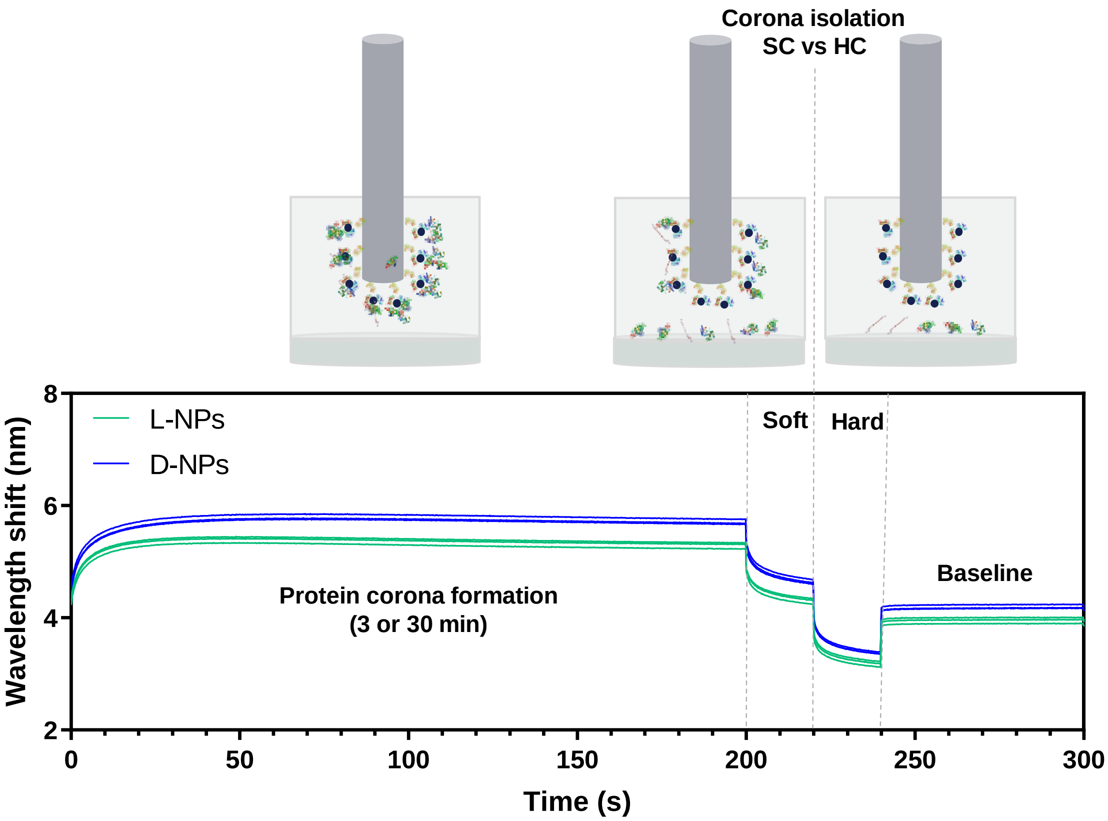


**Supplementary Figure 3. Schematic workflow of soft corona (SC) and hard corona (HC) isolation by Fishing method.** Chiral NP-loaded biosensors are firstly equilibrated into buffer solution for 1 min (not shown) with a further incubation in serum. 0.005% of WB and 0.1% of WB are chosen for the isolation of SC and HC, respectively. Collected proteins are measured by NanoDrop and 2 μg aliquots are collected and digested by trypsin for proteomics analysis. Source data are provided as a Source Data file.

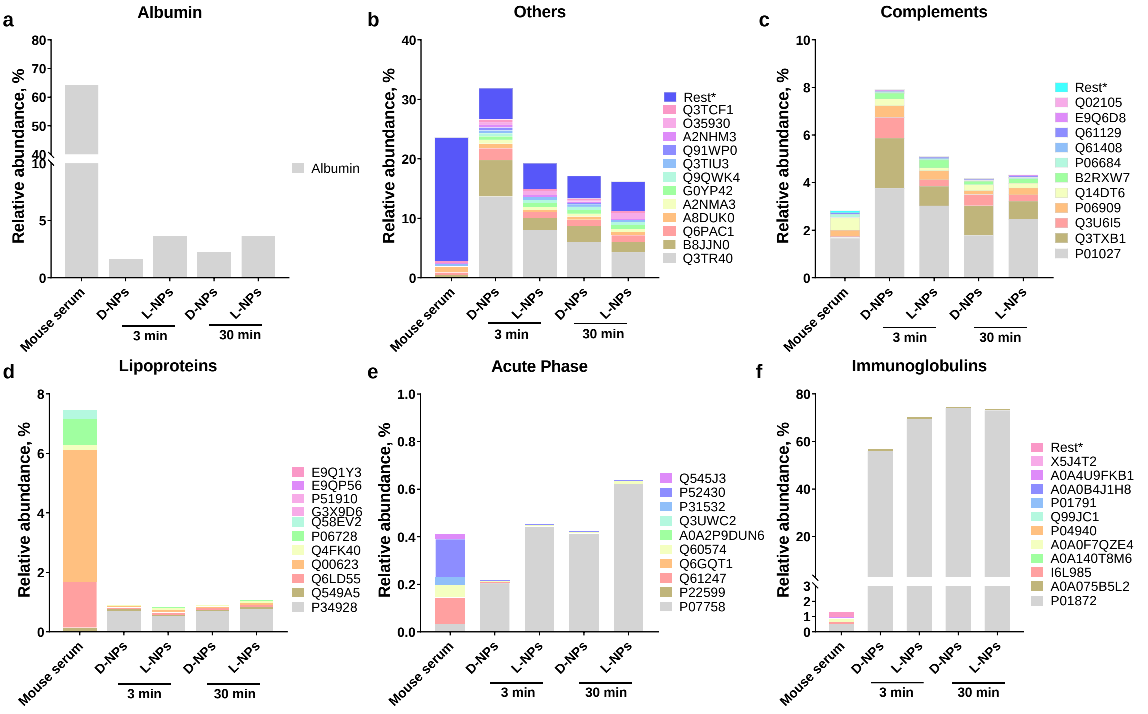


**Supplementary Figure 4. Analysis of protein abundance in mouse serum and hard corona components by centrifugation (HCC) formed on chiral NPs.** HCC components are obtained according to the centrifugation-based isolation upon 3 and 30 min incubation. Identified corona proteins (n = 3) are classified based on their biological function. Relative abundance of proteins classified as albumin (A), others (B), complements (C), lipoproteins (D), acute phase (E) and immunoglobulins (F). The relative abundance shown here represents three independent biological replicates for each sample. Proteomics data are presented in Supplementary Data file.

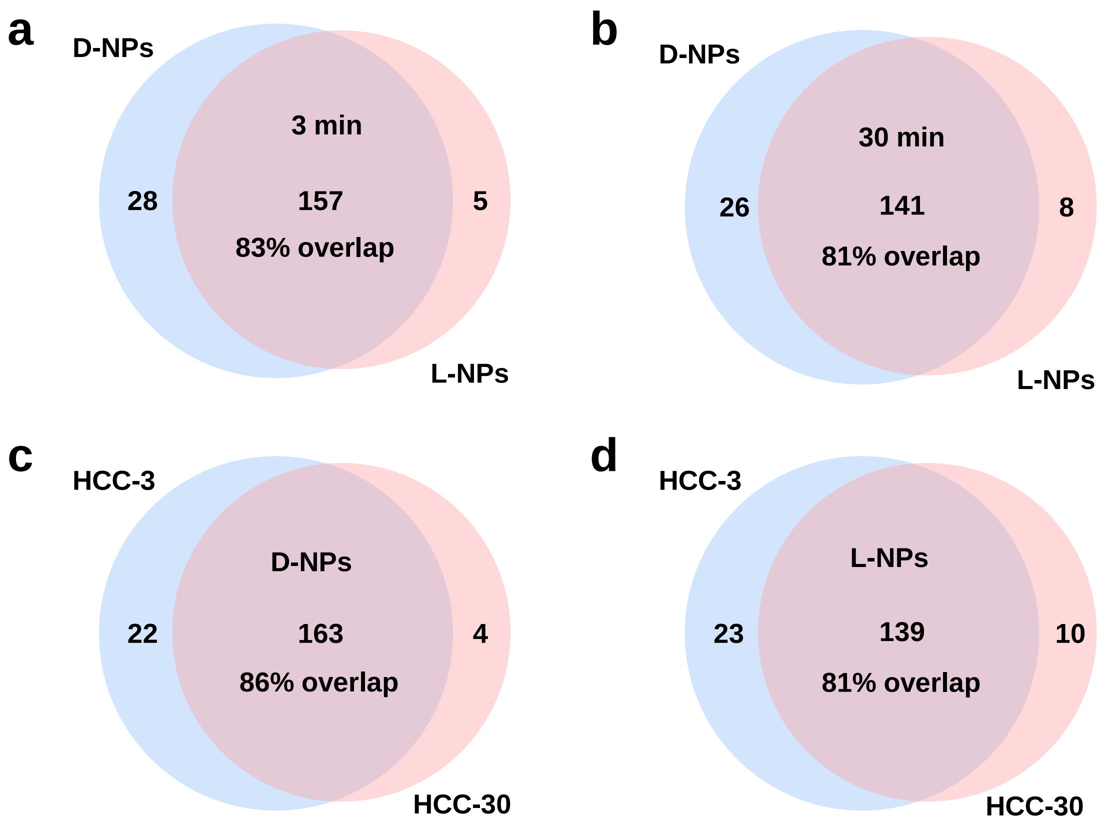


**Supplementary Figure 5. Analysis of time- and chirality-dependent HCCs formation**. Chirality-specific HCC on chiral NPs (n=3) after 3 min (HCC-3, a) and 30 min (HCC-30, b) incubation with mouse serum, respectively. Time-specific HCC evolution on D-NPs (c) and L-NPs (d). The data shown here represents three independent biological replicates for each sample. HCC-3, light blue color; HCC-30, light red color.

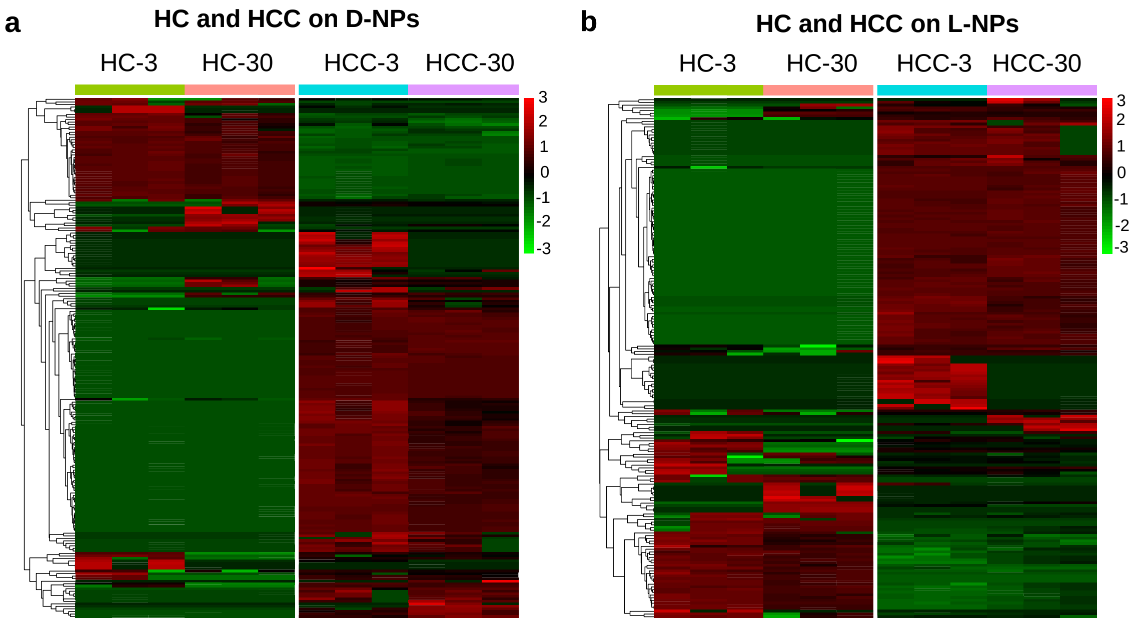


**Supplementary Figure 6. Heatmaps of the HCC and HC components.** a, b) Corona components isolated by the BLI-based Fishing method from the D-NPs (a) and L-NPs (b). HCC and HC of chiral NPs. Heatmaps show the median abundance of protein groups detected for two types of hard corona composition (columns) on chiral NPs (rows) at 3 min (HCC-3, HC-3) and 30 min incubation (HCC-30, HC-30). Data processing and cluster analysis are performed using R language (version 4.0.5). All data are acquired from triplicate biological replicates. HC-3, light green color; HC-30, i light red color. HCC-3, light blue color; HCC-30, light purple color.

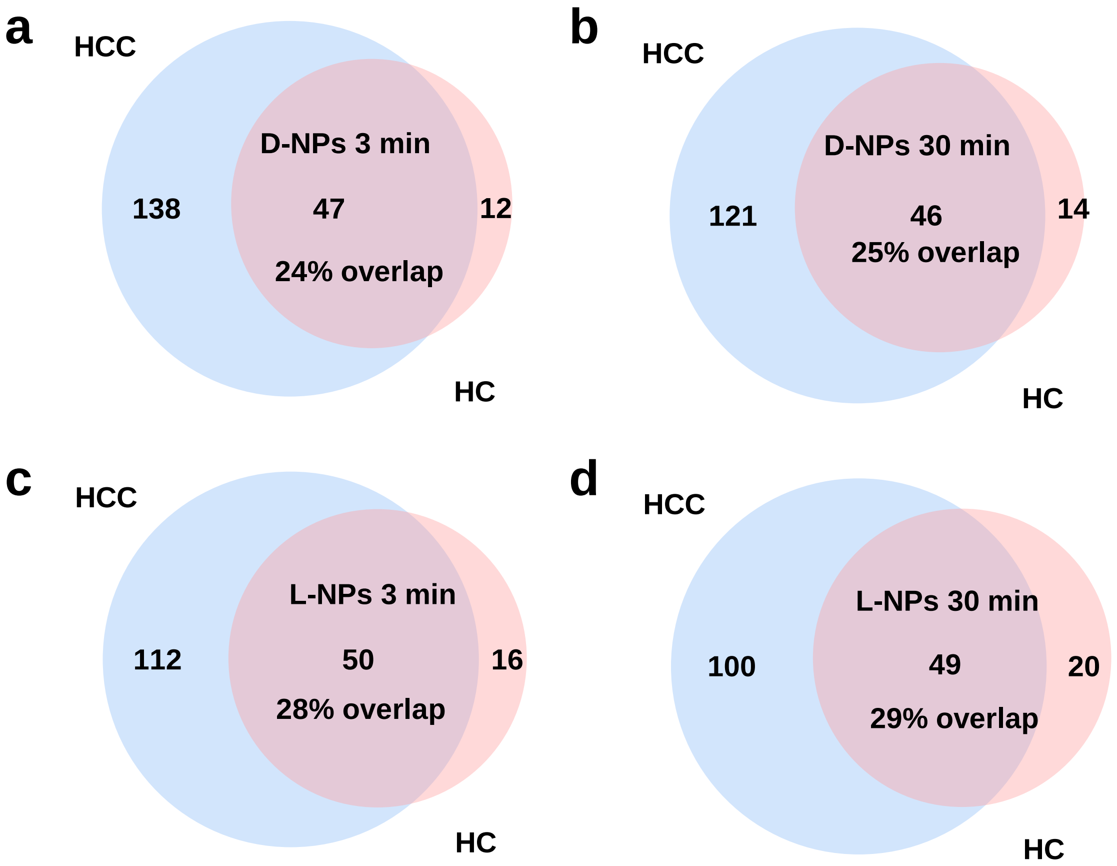


**Supplementary Figure 7. Composition similarity analysis of chiral HCC and HC proteins.** The composition of HCC and HC proteins on the surface of D-NPs and L-NPs after 3 min (a, c) and 30 min (b, d) incubation with 10% mouse serum, respectively. HCC, light blue color; HC, light red color. HCC, light blue color; HC, light red color.

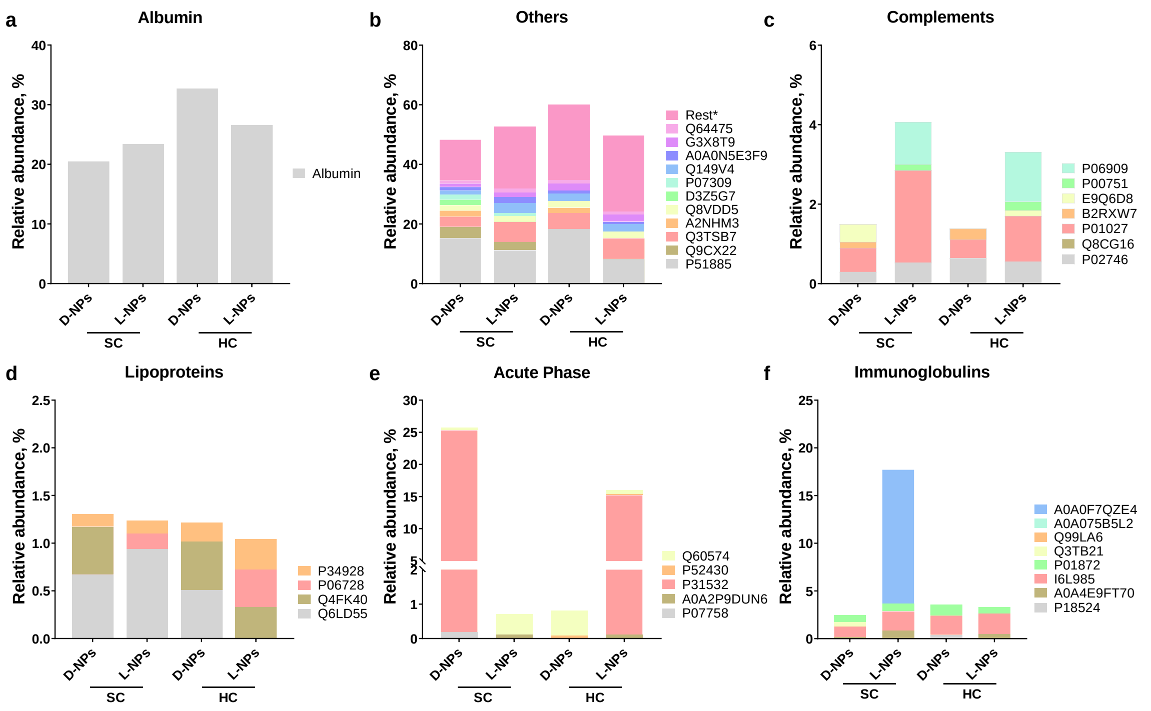


**Supplementary Figure 8. Major protein components in SC and HC layers on chiral NPs after 3 min interaction.** Identified corona proteins (n = 3) are classified based on their biological function. Relative abundance of major corona compositions is classified as albumin, others, complements, lipoproteins, acute phase, and immunoglobulins. The relative abundance shown here represents three independent biological replicates for each sample. Proteomics data are presented in Supplementary Data file.

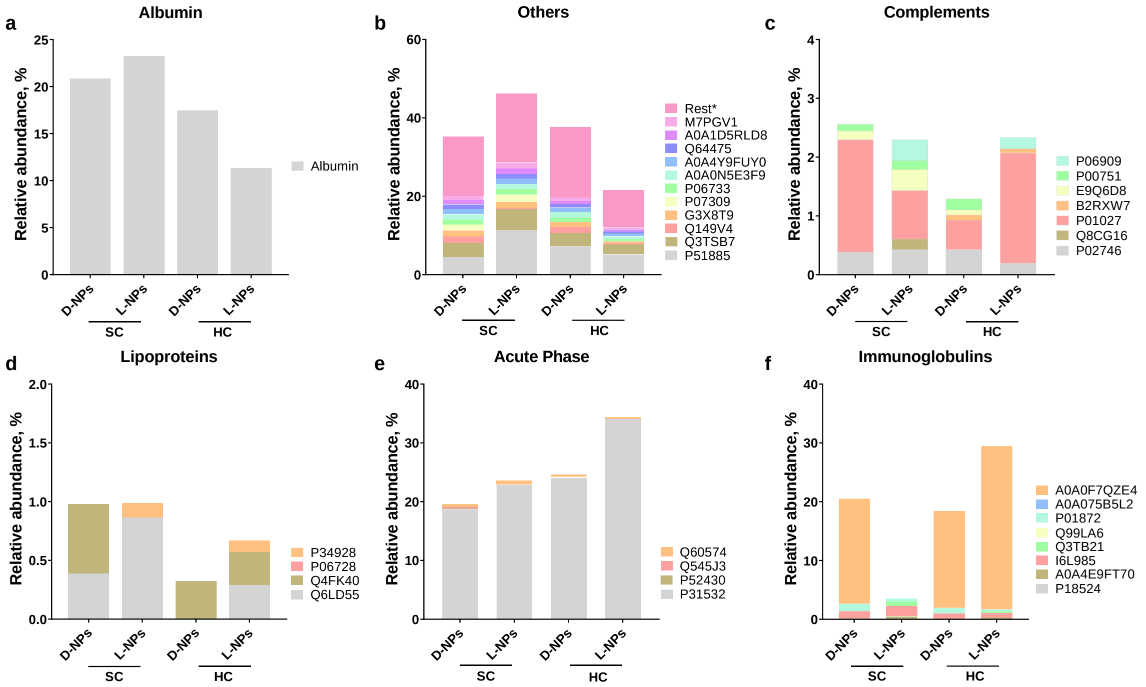


**Supplementary Figure 9. Major protein components in SC and HC layers formed on chiral NPs after 30 min interaction**. Identified corona proteins (n = 3) are classified based on their biological function. Relative abundance of major corona compositions is classified as albumin, others, complements, lipoproteins, acute phase, and immunoglobulins. The relative abundance shown here represents three independent biological replicates for each sample. Proteomics data are presented in Supplementary Data file.

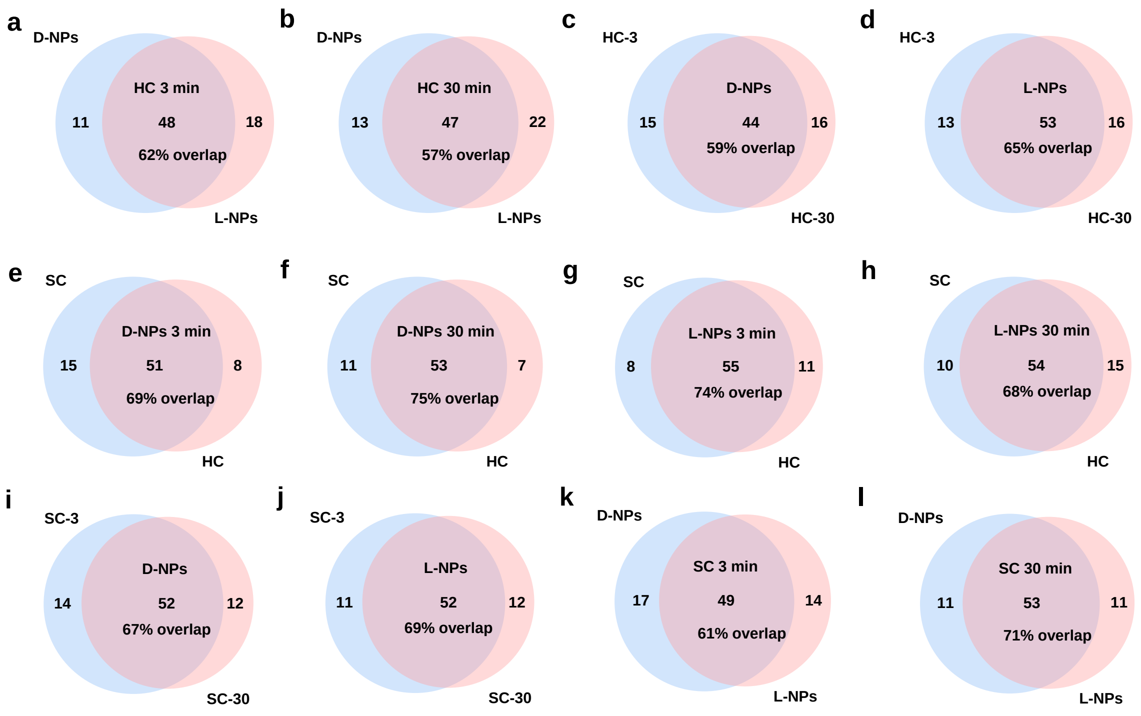


**Supplementary Figure 10. Analysis of protein composition similarity and its relationship with time and chirality.** Similarity in coronal composition for HC on chiral NPs at 3 min (a) and 30 min (b). Time-dependent similarity in HC composition on D-NPs (c) and L-NPs (d). Comparison of the SC and HC composition on D-NPs (e, f) and L-NPs (g, h) over time. Time-dependent similarity in SC composition on D-NPs (i) and L-NPs (j). Comparison of the SC compositions on chiral NPs at 3 min (SC-3, k) and 30 min (SC-30, l). D-NPs, SC, HC-3 and SC-3: light blue color; L-NPs, HC, HC-30 and SC-30: light red color.

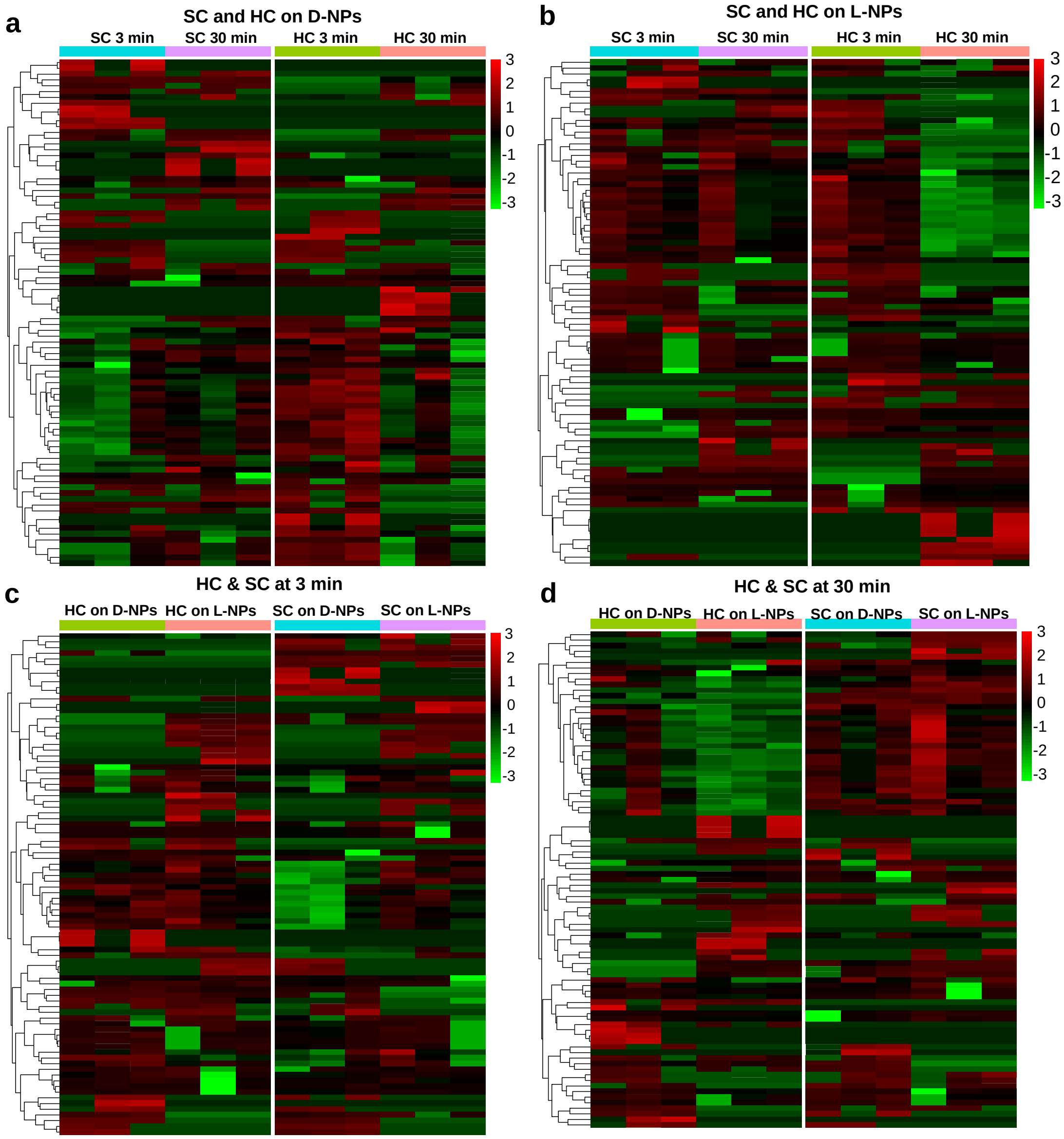


**Supplementary Figure 11. Heatmap of the SC vs HC composition on chiral NPs at different incubation time.** (a, b) Comparison of time-dependent hard corona (HC) with soft corona (SC) components on D-NPs (a) and L-NPs (b) at 3 and 30 min incubation. SC 3 min, light blue color; SC 30 min, light purple color; HC 3 min, light green color; HC 30 min, light red color. (c, d) Comparison of surface chirality-specific HC with SC components on NPs at 3 min (c) and 30 min (d) incubation. HC on D-NPs, light green color; HC on L-NPs, light red color; SC on D-NPs, light blue color; SC on L-NPs, light purple color. Corona components are isolated by the BLI-based Fishing method**.** Heatmaps show the median normalized abundance of protein groups detected for HC and SC protein composition (columns) on chiral NPs (rows). Data processing and cluster

analysis are performed using R language (version 4.0.5). All data are acquired from three biological replicates.

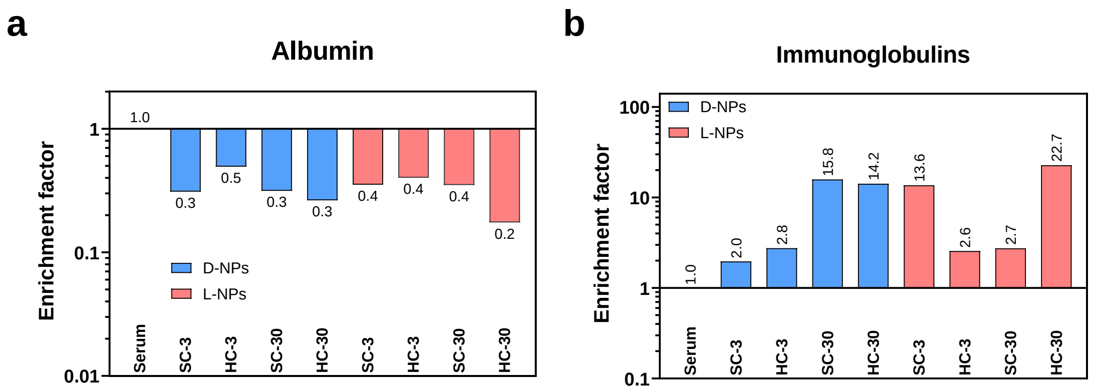


**Supplementary Figure 12. Enrichment factors for two representative proteins with high abundance.** Enrichment factor of serum albumin (a) and immunoglobulins (b) in SCs/HCs of chiral NPs over serum. Enrichment factor [(% in SC or HC)/(% in serum)] is calculated as relevant percentage of protein in corona per protein in 10% mouse serum. All data are acquired from triplicate biological experiments (n=3). Source data are provided as a Source Data file.

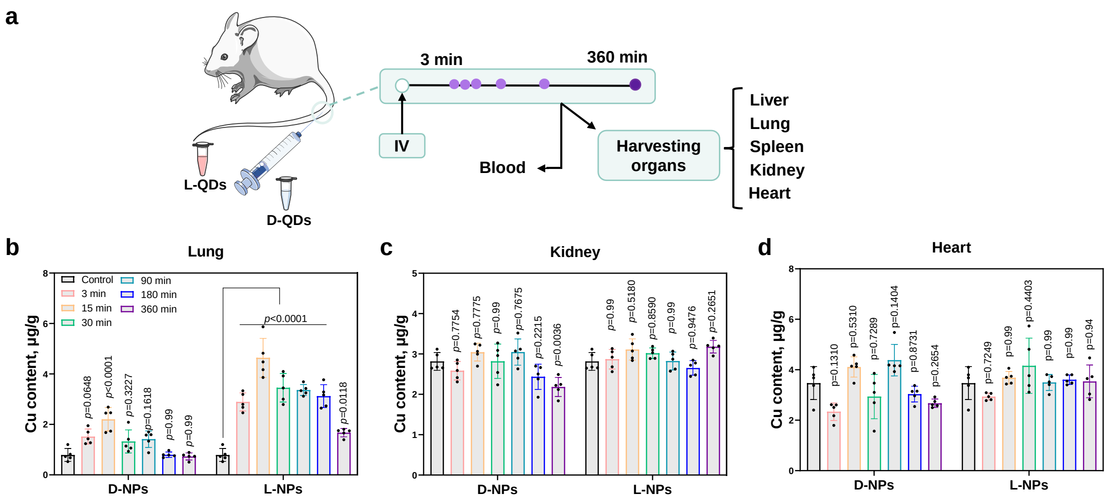


**Supplementary Figure 13. In vivo biodistribution of chiral NPs.** a) Overview of i.v. administered chiral NPs biodistribution in Balb/c mice (n = 5). Cu content in lung (b), kidney (c) and heart (d) is harvested and analyzed by ICP-MS to know the distribution of NPs. Statistical significance is calculated by two-way ANOVA with Tukey's multiple comparisons test. *$p < 0.05$; **$p < 0.01$; ***$p < 0.001$; ****$p < 0.0001$; n.s., not significant ($p > 0.05$). The data are shown as mean value and standard deviations for five biological replicates (n=5). Source data are provided as a Source Data file.

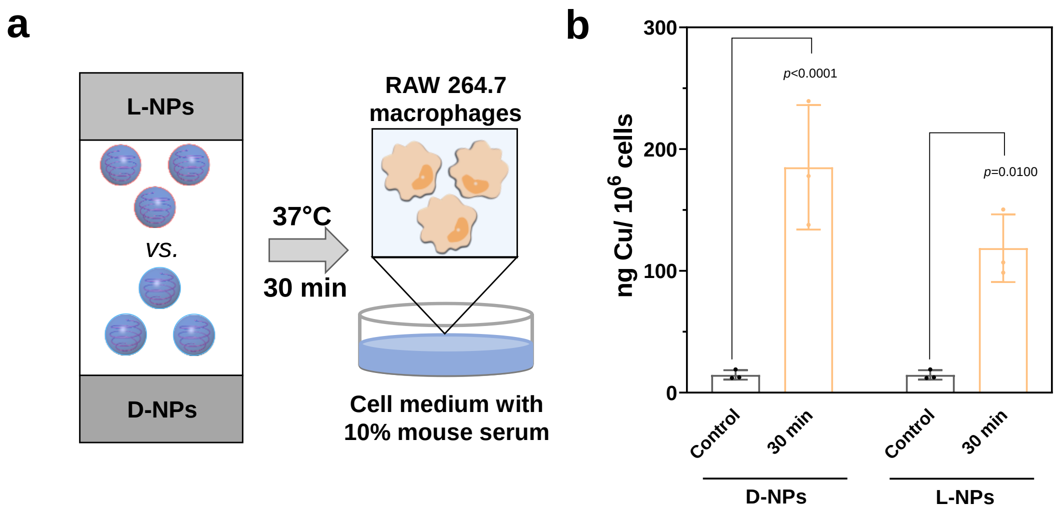


**Supplementary Figure 14. Effect of surface chirality on cellular uptake.** a) Schematic illustration of cellular uptake of chiral NPs. b) Cellular uptake of D-NPs and L-NPs in 10% mouse serum-supplemented cell culture medium within 30 min as determined by ICP-MS. Statistical significance is calculated by one-way ANOVA with Tukey's multiple comparisons test. *$p < 0.05$; ****$p < 0.0001$. Data are shown as mean value and standard deviations for triplicate biological samples (n=3). Source data are provided as a Source Data file.
