# Supplementary material for: In situ analysis of nanoparticle soft corona and dynamic evolution
Source: Nat Commun. 2022 Sep 14;13:5389. doi: 10.1038/s41467-022-33044-y (PMC9474507; doi:10.1038/s41467-022-33044-y)
Supplement: Supplementary file 3 — Description of Additional Supplementary Files [file 41467_2022_33044_MOESM3_ESM.docx]

**Description of Additional Supplementary Files**

File Name: Supplementary Data 1

Description: The details of the proteomics studies, including the information of the detected coronal proteins isolated by centrifugation and fishing approaches at different time points after incubation with mouse serum, and control biosensor samples and the list of most abundant proteins.
