# Supplementary material for: In situ analysis of nanoparticle soft corona and dynamic evolution
Source: Nat Commun. 2022 Sep 14;13:5389. doi: 10.1038/s41467-022-33044-y (PMC9474507; doi:10.1038/s41467-022-33044-y)
Supplement: Supplementary file 5 — Reporting Summary [file 41467_2022_33044_MOESM5_ESM.pdf]

nature portfolio

Corresponding author(s): Chunying Chen

Last updated by author(s): Aug 7, 2022

Reporting Summary

Nature Portfolio wishes to improve the reproducibility of the work that we publish. This form provides structure for consistency and transparency in reporting. For further information on Nature Portfolio policies, see our Editorial Policies and the Editorial Policy Checklist.

Statistics

For all statistical analyses, confirm that the following items are present in the figure legend, table legend, main text, or Methods section.

n/a	Confirmed	
☐	☒	The exact sample size (*n*) for each experimental group/condition, given as a discrete number and unit of measurement
☐	☒	A statement on whether measurements were taken from distinct samples or whether the same sample was measured repeatedly
☐	☒	The statistical test(s) used AND whether they are one- or two-sided<br>*Only common tests should be described solely by name; describe more complex techniques in the Methods section.*
☒	☐	A description of all covariates tested
☒	☐	A description of any assumptions or corrections, such as tests of normality and adjustment for multiple comparisons
☐	☒	A full description of the statistical parameters including central tendency (e.g. means) or other basic estimates (e.g. regression coefficient) AND variation (e.g. standard deviation) or associated estimates of uncertainty (e.g. confidence intervals)
☐	☒	For null hypothesis testing, the test statistic (e.g. *F*, *t*, *r*) with confidence intervals, effect sizes, degrees of freedom and *P* value noted<br>*Give P values as exact values whenever suitable.*
☒	☐	For Bayesian analysis, information on the choice of priors and Markov chain Monte Carlo settings
☐	☒	For hierarchical and complex designs, identification of the appropriate level for tests and full reporting of outcomes
☒	☐	Estimates of effect sizes (e.g. Cohen's *d*, Pearson's *r*), indicating how they were calculated

Our web collection on statistics for biologists contains articles on many of the points above.

Software and code

Policy information about availability of computer code

Data collection	LC-MS/MS data were obtained with Thermo Ultimate 3000 nano-UPLC system directly interfaced with a Thermo Fusion LUMOS mass spectrometer.<br>Bio-layer interferometry measurements were obtained with an Octet RED96e system (ForteBio, USA).<br>X-Ray Photoelectron Spectroscopy (XPS) was performed using a Thermo Scientific ESCALAB 250e III.<br>Transmission electron microscope (TEM) micrographs were obtained with a FEI Talos F200S (FEI Company, USA).<br>Circular dichroism (CD) spectra were obtained with a J-1500 CD spectrometer (Jasco, Japan).<br>X-ray diffraction (XRD) patterns were recorded on a STOE STADI MP diffractometer (STOE & Cie GmbH, Germany).<br>Induced Coupled Plasma Mass Spectrometry (ICP-MS) was obtained using a Thermo Elemental X7 ICP-MS.
Data analysis	GraphPad Prism Software (version 8.2.1, La Jolla California USA) was used for statistical data analysis and figure generation.<br>Image J (version 1.53k, NIH USA) was used to determine average size of NPs based on TEM images.<br>Octet Data analysis software (ForteBio, version 11.0) was used to detect of protein corona formation, isolation and to analyze proteins association and dissociation rate.<br>Proteome Discoverer (Thermo Scientific, version 2.4) was used for proteomics data analysis.<br>Raw data were manually assigned via searching all entries from the mouse on Uniprot.org database and known contaminants.<br>Proteins were generated, color modified, and visualized by PyMOL Molecular Graphics System (version 2.2).<br>X-ray photoelectron spectra were processed with XPS Peak 4.1.<br>Proteomics data processing and cluster analysis were performed by R language (version R 4.0.5).<br>Fusion LUMOS mass spectrometer was operated in data-dependent acquisition mode using Xcalibur (version 4.1.50) software.

For manuscripts utilizing custom algorithms or software that are central to the research but not yet described in published literature, software must be made available to editors and reviewers. We strongly encourage code deposition in a community repository (e.g. GitHub). See the Nature Portfolio guidelines for submitting code & software for further information.

Data

Policy information about availability of data

All manuscripts must include a data availability statement. This statement should provide the following information, where applicable:

- Accession codes, unique identifiers, or web links for publicly available datasets
- A description of any restrictions on data availability
- For clinical datasets or third party data, please ensure that the statement adheres to our policy

The data generated in this study is available with the article, Supplementary information, and Supplementary data. All other data are available from the corresponding authors upon request. The mouse proteomic dataset is available on the UniProt website (https://www.uniprot.org). The proteomic data generated by mass spectrometry, including raw data and search results have been deposited to the ProteomeXchange Consortium (http://proteomecentral.proteomexchange.org) via the PRIDE partner repository with the dataset identifier: PXD033976 [http://proteomecentral.proteomexchange.org/cgi/GetDataset?ID=PXD033976].55 PDB files 1SI4 [http://doi.org/10.2210/pdb1SI4/pdb], 1AO6 [http://doi.org/10.2210/pdb1AO6/pdb], 1IGY [http://doi.org/10.2210/pdb1IGY/pdb], 3GHG [http://doi.org/10.2210/pdb3GHG/pdb] and 1D3K [http://doi.org/10.2210/pdb1D3K/pdb] were obtained from the RCSB Protein Data Bank (PDB) www.rcsb.org. The identified coronal protein parameters, including molecular weight, isoelectric points and relative abundance (in Supplementary Figs. 4, 8, 9 and Figs. 2b, 3b, 4b) generated in this study are presented in Supplementary Data file. Data processing and cluster analysis are performed using R language (version 4.0.5). Protein abundance is normalized to reduce the effect of outliers and minimize overall proteome difference. Hierarchical clustering analysis is performed by the R package heatmap on the normalized, log2 abundance using Euclidean distance. Illustrations of tubes, three neck flasks, mouse and syringe in Fig. 2a, Supplementary Figs. 1a and 13a were obtained from SMART- servier medical art (https://smart.servier.com/). The source data including Figs. 1e-h, 2b, 3b, 4b, 5b, 5c, 5e, 5f, 5h, 5i, 5j-l, and Supplementary Figs. 1b-d, 2, 3, 12, 13b-d, 14b are provided in a Source Data file. The source data are provided with this paper.

Field-specific reporting

Please select the one below that is the best fit for your research. If you are not sure, read the appropriate sections before making your selection.

☒ Life sciences ☐ Behavioural & social sciences ☐ Ecological, evolutionary & environmental sciences

For a reference copy of the document with all sections, see nature.com/documents/nr-reporting-summary-flat.pdf

Life sciences study design

All studies must disclose on these points even when the disclosure is negative.

Sample size	The sample size used for statistical is indicated for each experiment. Sample size is indicated in the figure legends for each experiment. A minimum sample size of n=3 was used. Sample size was determined based on preliminary experiments and previous similar work:<br>For animal experiments, refer to Nature Nanotechnology, 16, 708–716 (2021);<br>For the cell uptake experiment, refer to ACS Nano, 14, 5529–5542 (2020);<br>For the proteomic experiments, refer to Nature Nanotechnology, 16, 708–716 (2021);<br>For BLI experiments, refer to Acta Pharmaceutica Sinica B, 12, 1723-1739 (2022).
Data exclusions	No data were excluded from the method analysis.
Replication	Experiments were replicated independently with successful replications. In details, proteomic experiments were conducted with three biological replicates. For in vitro cellular uptake study, cell sample preparation was done with three biological replicates and ICP-MS measurement for each sample was done with three technical replicates. For bio-distribution study, the experiments were conducted for five biological replicates and ICP-MS measurement for each sample was done with three technical replicates.
Randomization	For all in vitro experiments, cultured cells were randomly assigned to experimental groups. For the animal study, mice were randomly allocated to each group before treatment.
Blinding	The investigator was blinded to the group allocation during data collection and analysis.

Reporting for specific materials, systems and methods

We require information from authors about some types of materials, experimental systems and methods used in many studies. Here, indicate whether each material, system or method listed is relevant to your study. If you are not sure if a list item applies to your research, read the appropriate section before selecting a response.

Materials & experimental systems

n/a	Involved in the study	
☒	☐	Antibodies
☐	☒	Eukaryotic cell lines
☒	☐	Palaeontology and archaeology
☐	☒	Animals and other organisms
☒	☐	Human research participants
☒	☐	Clinical data
☒	☐	Dual use research of concern

Methods

n/a	Involved in the study	
☒	☐	ChIP-seq
☒	☐	Flow cytometry
☒	☐	MRI-based neuroimaging

Eukaryotic cell lines

Policy information about cell lines

Cell line source(s)	Murine RAW 264.7 macrophages (catalog number SCSP-5036) were purchased from National Collection of Authenticated Cell Cultures (Shanghai, China).
Authentication	Not authentication for this study.
Mycoplasma contamination	Negative for Mycoplasma contamination.
Commonly misidentified lines (See ICLAC register)	No commonly misidentified cell lines were used in the study.

Animals and other organisms

Policy information about studies involving animals; ARRIVE guidelines recommended for reporting animal research

Laboratory animals	Pathogen-free BALB/c mice were purchased from Beijing Vital River Laboratory Animal Technology. All the animals were maintained on a standard diet and water ad libitum at 22±2 °C and 50–60% relative humidity on a 12 h light/12 h dark cycle. Female BALB/C mice (n = 5, 6-8 weeks) were used in in vivo bio-distribution and proteomics studies.
Wild animals	No wild animals were used in the study.
Field-collected samples	This study did not involve field-collected samples.
Ethics oversight	All protocols were approved by the Institutional Animal Care and Use Committee of National Center for Nanoscience and Technology and performed under the ethical guidelines for the use and care of animals.

Note that full information on the approval of the study protocol must also be provided in the manuscript.
